# Quantification of Ligand Binding to G-Protein Coupled Receptors on Cell Membranes by Ellipsometry

**DOI:** 10.1371/journal.pone.0046221

**Published:** 2012-09-26

**Authors:** Verena Kriechbaumer, Alexei Nabok, Robert Widdowson, David P. Smith, Ben M. Abell

**Affiliations:** 1 Biomedical Research Centre, Sheffield Hallam University, Sheffield, United Kingdom; 2 Materials and Engineering Research Institute, Sheffield Hallam University, Sheffield, United Kingdom; University of Iowa, United States of America

## Abstract

G-protein-coupled receptors (GPCRs) are prime drug targets and targeted by approximately 60% of current therapeutic drugs such as β-blockers, antipsychotics and analgesics. However, no biophysical methods are available to quantify their interactions with ligand binding in a native environment. Here, we use ellipsometry to quantify specific interactions of receptors within native cell membranes. As a model system, the GPCR-ligand CXCL12α and its receptor CXCR4 are used. Human-derived Ishikawa cells were deposited onto gold coated slides via Langmuir-Schaefer film deposition and interactions between the receptor CXCR4 on these cells and its ligand CXCL12α were detected via total internal reflection ellipsometry (TIRE). This interaction could be inhibited by application of the CXCR4-binding drug AMD3100. Advantages of this approach are that it allows measurement of interactions in a lipid environment without the need for labelling, protein purification or reconstitution of membrane proteins. This technique is potentially applicable to a wide variety of cell types and their membrane receptors, providing a novel method to determine ligand or drug interactions targeting GPCRs and other membrane proteins.

## Introduction

Eukaryotic cells depend on compartmentalization of proteins and metabolites for their biochemical processes and membrane-associated signalling is fundamental to cellular regulation. The importance of studying membrane proteins is highlighted by the fact that almost half of the top-selling drugs target membrane proteins [1]. However, monitoring protein interactions in lipid environments is technically challenging. Existing biochemical and biophysical methods to study membrane-protein interactions require isolation or recombinant expression of the protein followed by reconstitution into a lipid environment, which presents issues of purification, yield and correct protein folding. Hence few techniques are available to study specific membrane-protein interactions *in situ*.

**Figure 1:**
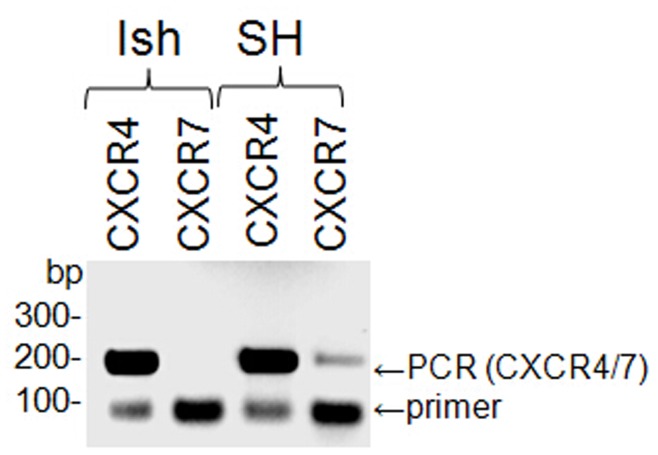
Detection of CXCR4 and CXCR7 mRNA in cell lines. Reverse transcriptase PCR was performed using primer combinations specific for CXCR4 and CXCR7, respectively, with RNA isolated from Ishikawa (Ish) cells as well as from the neuronal cell line SH-SY5Y (SH). PCR-fragment sizes are indicated in basepairs (bp) on the left.

### G-protein Coupled Receptors

A structural class and the largest family of integral membrane proteins are G-protein-coupled receptors (GPCRs). Their diversity and importance in cellular signalling makes them prime drug targets. GPCRs are targeted by approximately 60% of all therapeutic drugs and 25% of the top-selling drugs [2] such as β-blockers, antipsychotics and analgesics. Well known examples for drugs against GPCRs are the antihistamine Clarinex (Schering-Plough), the ulcer treatment Zantac (GlaxoSmithKline) or Zyprexa (Eli Lilly) which is used to treat the symptoms of psychotic conditions such as schizophrenia and bipolar disorder [3]. GPCR malfunction is also involved in various diseases such as diabetes, obesity, cancer, hypothyroidism and psychotic disorders [4]. The G-protein coupled receptor (GPCR) superfamily includes receptors for a variety of different ligands such as hormones, neurotransmitters and inflammatory mediators. They all feature a structure of seven α-helical transmembrane domains with an extracellular N-terminus and cytosolic C-terminus [5], [6]. Various findings support the idea that GPCRs are present as homo-or heterodimers or oligomers via their transmembrane domains, leading to altered functionality and ligand pharmacology [7]. This highlights the need to investigate GPCRs in their native membrane environment.

**Figure 2:**
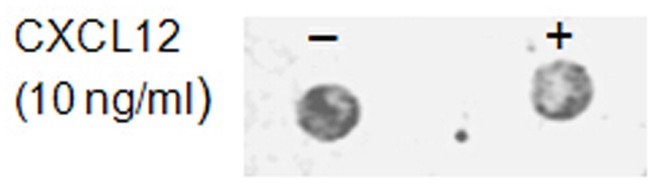
Effect of CXCL12α on the presence of CXCR4. Ishikawa cells in Tris-buffer were incubated with and without the chemokine CXCL12α. The cells were then spotted on a nitrocellulose membrane and immunoblots with anti-CXCR4 IgGs were performed.

### Langmuir-Schaefer Deposition

Despite the biological significance of GPCRs, their study has been limited by the lack of experimental systems mimicking their natural environment. Membrane receptors are difficult to express and purify in large quantities due to the instability of hydrophobic membrane spans in aqueous buffers. Commonly used methods to stabilise membrane proteins include the use of detergent micelles, bicelles or liposomes. Historically, detergents were used to form detergent-protein-lipid micelles, but detergent can decrease protein stability, interfere with assays and can cause partitioning of substrates and products into the excess detergent micelle.

In contrast to detergent micelles, liposomes can be composed of specific types of phospholipids required by many membrane protein systems to maintain active function. Incorporation of membrane proteins into liposomes is useful when compartmentalisation is required, such as in ion channel assays. However liposomes are large, unstable and difficult to prepare at a controlled size and stoichiometry [8]. Furthermore for presenting receptors in synthetic lipid layers, receptor function has to be reconstituted [9]. More recently, reconstituted high-density lipoprotein particles (rHDL) were used for packaging transmembrane proteins in a native-like membrane environment, allowing solubilisation and delivery of hydrophobic drugs. In this system, membrane proteins in detergent micelles are reconstituted into lipid bilayers of apolipoprotein particles. These particles are homogenous and monodispersed with receptors incorporated in monomeric form. This system has been tested for isolated membrane proteins such as bacteriorhodopsin [8], and the protective antigen pore of the anthrax toxin [10].

**Figure 3:**
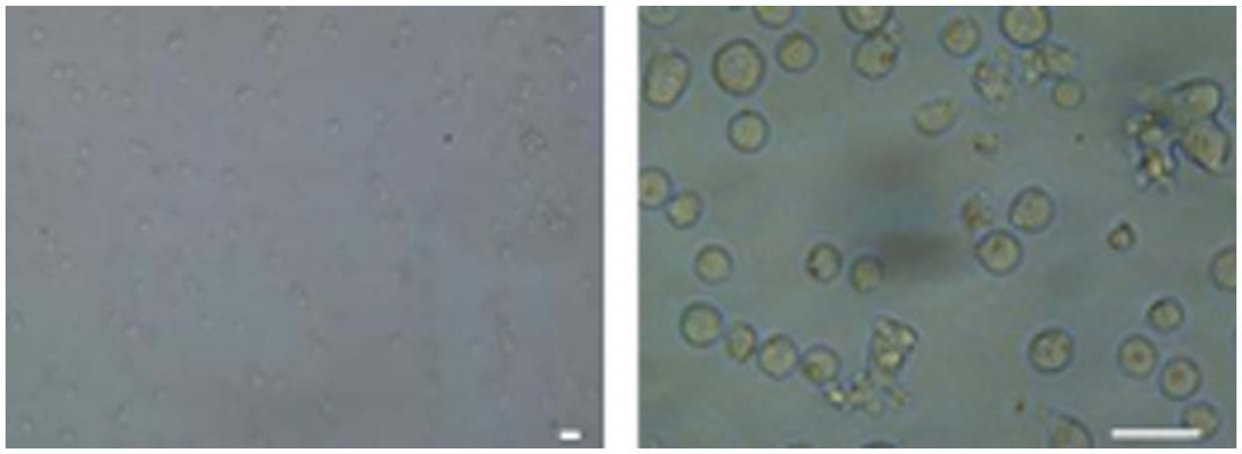
Microscopy of Ishikawa cells after preparation for TIRE. Ishikawa cells snap-frozen in Tris-buffer were thawed and membrane intactness was observed using a light microscope at 20× (left) and 40× (right) magnification. Bar = 50 µm.

Another approach to analyse membrane proteins is to overexpress them in cell lines, but this typically requires a non-native cell line that has different membrane constituents, and will distort interactions that depend on a natural abundance of receptors. Therefore, we used a novel technique involving Langmuir-Schaefer deposition of whole cells, resulting in receptor proteins being displayed in a thin film in their native environment. Interactions could then be monitored by changes in layer thickness.

### TIRE Method

An effective system for quantifying protein interactions is surface plasmon resonance (SPR), which has been adapted for membrane interactions by using supported bilayers or lipid vesicles [11]. However, difficulties can arise from the membrane deposition step, and from a lack of sensitivity due to the low density of target receptors at native membranes. To address these problems with SPR we combined Langmuir-Schaefer deposition with total internal reflection ellipsometry (TIRE) to generate sufficient sensitivity, an approach that has previously been successfully used to quantify membrane receptor interactions at the surface of chloroplasts [12]. The spectroscopic method of TIRE combines ellipsometry and SPR to detect changes in the polarization of reflected light by measuring the two parameters Ψ and Δ related to the amplitude and the phase of polarized light, respectively. TIRE has been used to analyze interactions between antibodies and various ligands [13], [14] including the analysis of pesticides and mycotoxins with detection levels as low as 0.1 ng/ml [15].

**Figure 4:**
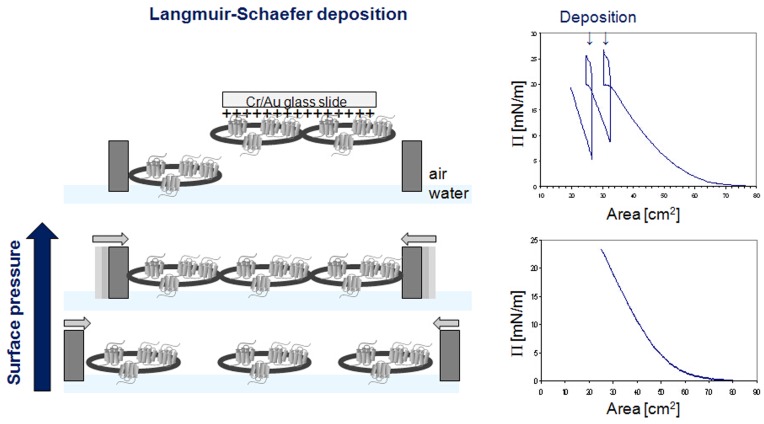
Langmuir-Schaefer deposition of human cells. Cell droplets were spread over the water surface in the Langmuir trough, and the trough barriers were moved towards each other to compress the cells; the surface pressure diagrams are shown on the right hand side. At a surface pressure Π of 20 mN/m - just before pressure saturation when cells are present in a uniform layer - Cr/Au coated glass slides carrying a positive charge were brought in to contact with the cells on the water surface and removed again, transferring a portion of the cell layer onto the slide.

As a model system the interaction between the chemokine CXCL12α and cells natively expressing its GPCR CXCR4 was tested. CXCR4 was first identified as a coreceptor for HIV-1 entry into cells [16], but also functions in cancer metastasis [17]. Recent reports show that CXCL12α also binds the GPCR receptor CXCR7 [18], although this interaction does not induce the typical chemokine responses of cell migration and intracellular signal transduction [19], [20], [18]. It is known that the initial interaction between CXCL12α and CXCR4 takes place between the residues 12 to 17 of CXCL12α and extracellular N-terminal residues 2–36 of CXCR4 [21], [22]. We show here that TIRE can be used to obtain binding affinities of specific chemokine-receptor interactions on cell membranes without the need to purify or overexpress the target receptor protein. Binding can be inhibited by application of the CXCR4-binding drug AMD3100, showing the specificity of the detected interaction.

## Materials and Methods

### Ishikawa Cell Line Culture

All cell culture methods were carried out under sterile conditions in a class II laminar flow cabinet. Ishikawa endometrial adenocarcinoma cells and the neuronal cells SH-SY5Y were obtained from the European Collection of Cell Cultures. Thawed stocks of adherent cells were routinely grown and passaged in a cell culture incubator at 37°C and 5% CO_2_ in 25 cm^2^ cell culture flasks bathed in 20 ml of DMEM (Dulbecco’s Modified Eagle’s Medium supplemented with 2% (v/v) L-alanyl-L-glutamine (GIBCO® GlutaMAX™) and 2% penicillin-streptomycin (Invitrogen)). Cell monolayers were determined to be near-confluent at approximately 80%. The cells were detached from the flask by incubating in 5 ml of trypsin/EDTA solution (containing 0.25% trypsin and 0.02% EDTA) at 37°C for 5 min. Trypsin digestion was stopped by addition of 5 ml of supplemented DMEM. The resulting cell solution was placed into 50 ml Falcon tubes and centrifuged for 5 min at 300×g and the supernatant discarded. Cell pellets were washed twice by re-suspension and centrifugation in 10 ml of PBS, and the pellet was re-suspended in 8 ml of DMEM. 1 ml of the cell solution was passaged into new 25 cm^2^ flasks with 19 ml of DMEM. The remaining cells were pelleted again for 5 min at 300×g, and resuspended in 100 mM Tris-HCl, pH 8.0, aliquotted, snap-frozen in liquid nitrogen and stored at −80°.

**Figure 5:**
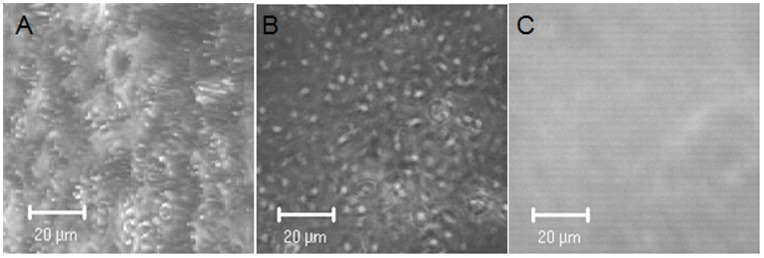
Cell deposition on Cr/Au coated glass slides. Confocal stacked images in phase contrast mode were taken from glass slides coated with Cr/Au with (A, B) and without (C) Langmuir-Schaefer cell deposition. A) shows the cell stack at a 30° angle.

### Immunoblotting

5 µl of whole Ishikawa cells or bovine serum albumin (BSA at 1 µg/µl) were spotted on Amersham Hybond™-N nylon blotting membranes (GE Healthcare). The membranes were blocked for 1 h at RT with 5% milk powder in Tris-Salt-Tween buffer (TST: 50 mM Tris-HCl, 150 mM NaCl, 0.5% (v/v) Tween 20, pH 8.0). After 1 h incubation with anti-CXCR4 IgG (Sigma) at a 1∶2000 dilution in TST, three washes with TST for 15 min each were performed. These primary antibodies were raised against a peptide corresponding to aa 182–196 in the second extracellular loop of human CXCR4 and therefore should not show competitive binding with the chemokine CXCL12α binding to the receptor N-terminus. The secondary goat anti-rabbit IgG labelled with red-fluorescent Alexa Fluor® 594 dye (Invitrogen) was used at a dilution of 1∶3000 in TST for 1 h and after three 5 min washes in TST signals were detected using the ODYSSEY Infrared imaging system (LI-COR Biosciences).

**Figure 6:**
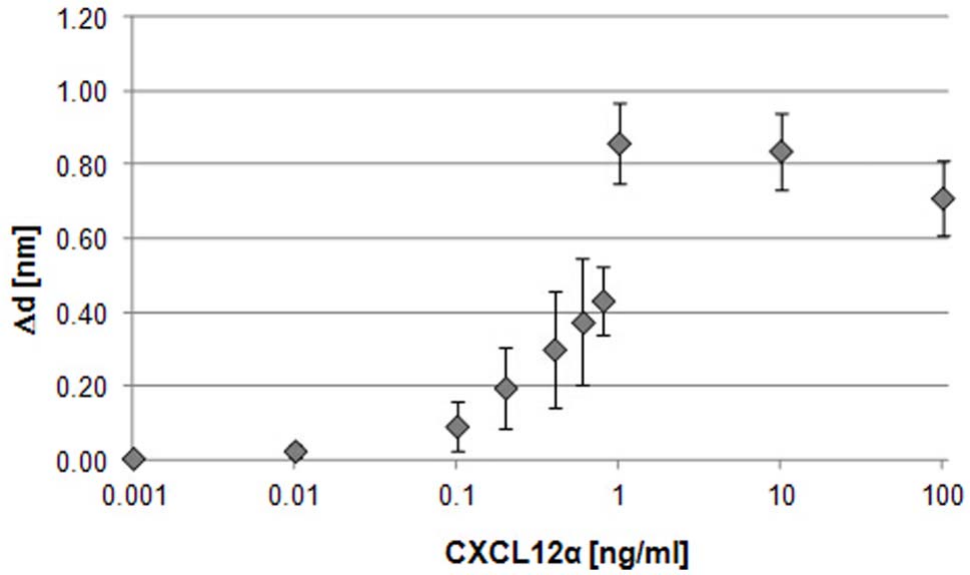
CXCL12α binding to receptors at the cell membrane of Ishikawa cells. Binding of the chemokine CXCL12α to Ishikawa cells was analysed by TIRE. Calibration curves (layer thickness increment Δd vs. increasing chemokine concentrations in ng/ml) show binding to native cell membranes deposited on a gold surface. n = 5. Standards errors are shown.

### Reverse Transcriptase-PCR Analysis

RNA from cells was isolated using TRIzol® reagent (Invitrogen) according to the manual, and the isolated RNA was treated for 15 min at room temperature with RNase-free DNaseI (Invitrogen) at 0.2 U/µg RNA (Invitrogen). cDNAs were created using the ProtoScript® AMV First Strand cDNA synthesis kit (New England Biolabs) according to the manufacturers’ instructions using random primers. PCR was performed using the following primers in 30 cycles with Phusion® High-Fidelity DNA Polymerase (New England Biolabs) according to the manufacturers’ suggestions with an annealing temperature of 55°C: for CXCR4 the forward primer 5′-CAGCAGGTAGCAAAGTGACG-3′ and the reverse primer 5′-GTAGATGGTGGGCAGGAAGA-3′ yield a 208-bp product; for CXCR7 the forward primer 5′-GCAGAGCTCACAGTTGTTGC-3′ and the reverse primer 5′-GCTGATGTCCGAGAAGT TCC-3′ yield a 189-bp product.

**Figure 7:**
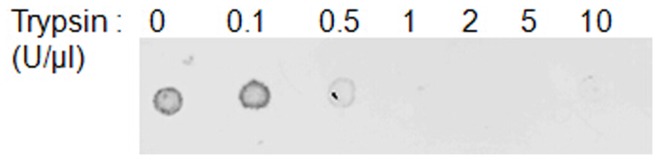
Trypsin treatment of cells. Ishikawa cells were treated with the protease trypsin in increasing concentrations up to 10 U/µl for 20 min, which was then inhibited using soybean trypsin inhibitor 1 mg/ml for 15 min. Cells were tested for the presence of the receptor CXCR4 by immunoblotting.

### Langmuir-Schaefer Deposition

Langmuir-Schaefer deposition was essentially performed as described previously in detail for chloroplast membranes [12]. Briefly, microscopic glass slides (1′′×1′′) were coated with Cr (3 nm thick) and Au (25 nm thick) using a thermal evaporation unit (Edwards A360) under a vacuum of 10^−6^ Tor. Slides were incubated overnight in 100 mM cysteamine-HCl, and deposition was performed in a Langmuir trough (KSV NIMA, Espoo, Finland). Membrane layers (Ishikawa cells or SH-SY5Y, respectively) were created on the surface of de-ionized water by dotting 300 µl of the cell solution with a Hamilton syringe. Deposition was performed at a surface pressure of 20 mN/m, and membranes were transferred onto slides by horizontal lifting [23].

**Figure 8:**
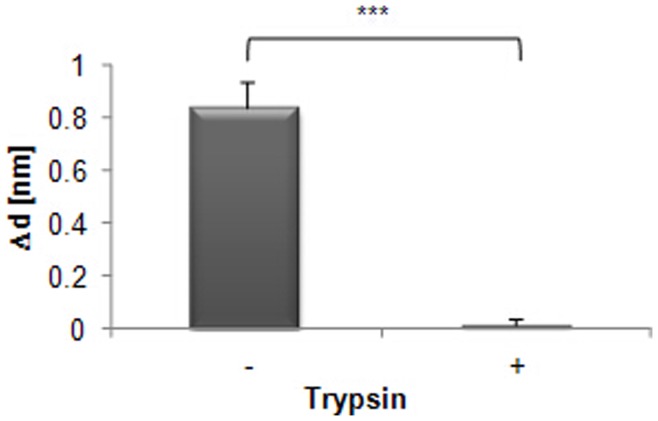
CXCL12α binding to Ishikawa cells analysed by TIRE after trypsin treatment. Increase in layer thickness Δd after addition of 10 ng/ml CXCL12α is shown (−) and compared to binding of the ligand after trypsin treatment (+) of the Ishikawa cells. Significant differences in binding are indicated as follows (n = 3): *** = p≤0.001. Standards errors are shown.

### TIRE Measurements and Data Fitting

TIRE measurements and fitting were also performed as described in [12] using ligand concentration from 1 pg/ml to 100 ng/ml. In brief, TIRE measurements were performed with the J.A. Woollam spectroscopic ellipsometer M2000 operating in the spectral range of 370–1000 nm with a rotating compensator. Index matching fluid was applied to obtain optical contact between the prism and glass slide. The reaction chamber with a volume of 0.2 ml was positioned underneath the cell layer of the slide. Cr/Au slides were washed with 20 chamber volumes of 100 mM Tris-HCl, pH 8.0, before flushing the reaction chamber with ligands in 100 mM Tris-HCl, pH 8.0 in increasing concentrations (from 1 pg/ml to 100 ng/ml) allowing 10 min incubation time. Wash steps with 20 volumes of 100 mM Tris-HCl, pH 8.0 were included after every incubation step. For proteolytic digestion cells were incubated with 1 U/µl trypsin (Sigma, Dorset, UK) for 15 min at 20°C. Before addition of ligands trypsin was inactivated by applying soybean trypsin inhibitor (Sigma, Dorset, UK) at 1 mg/ml for 15 min. To determine binding specificity AMD3100 octahydrochloride hydrate (Sigma, Plerixafor), a known inhibitor of the CXCL12α binding to CXCR4, was also used in TIRE measurements. Luteinizing hormone releasing hormone (LH-LR; Sigma, Dorset, UK) and follicle stimulating hormone (FSH; Sigma, Dorset, UK) were included in TIRE-binding experiments to Ishikawa cells at concentrations of 100 ng/ml.

**Figure 9:**
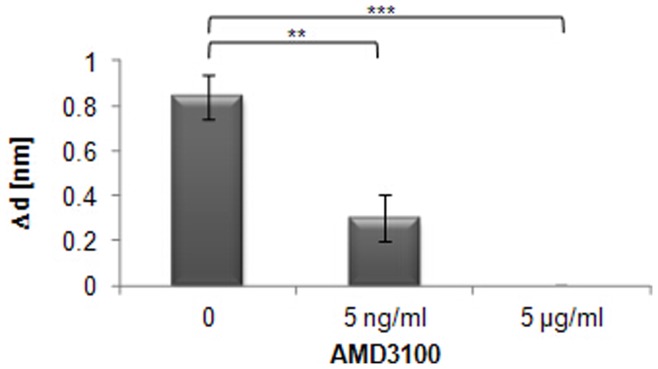
Inhibition by AMD3100 of CXCL12α binding to Ishikawa cells analysed by TIRE. Ishikawa cells were incubated with various concentrations of the inhibitor AMD3100 as indicated, followed by incubation with the ligand CXCL12α. Significant differences in binding are indicated as follows (n = 3): *** = p≤0.001; ** = p≤0.01. Standards errors are shown.

Spectroscopic ellipsometry provides the spectra of two ellipsometric parameters Ψ and Δ, which are related to the ratio of the amplitudes and the phase shift of p- and s- components of polarized light, respectively. Two types of ellipsometric measurements were performed: (I) TIRE single spectra scans were recorded after every adsorption step in a standard buffer solution (100 mM Tris-HCl, pH 8.0); and (II) dynamic TIRE spectral measurements, recording a number of TIRE spectra during adsorption. Dynamic TIRE measurements are used to analyze the kinetics of adsorption. Single spectra scans performed in the same buffer solution in steady-state conditions after completion of adsorption are suitable for TIRE data fitting. Software provided by J.A. Woollam Ltd [24] allowed the modelling of the reflection system and subsequent evaluation of the thickness and refractive index of adsorbed molecular layers by comparing the experimental and theoretical values of Ψ and Δ [12]. P-values were calculated using a Mann-Whitney U-test. For the evaluation of association (or affinity) constants a standard procedure of adsorption kinetics [25], [26] was used as described in [12].

**Figure 10:**
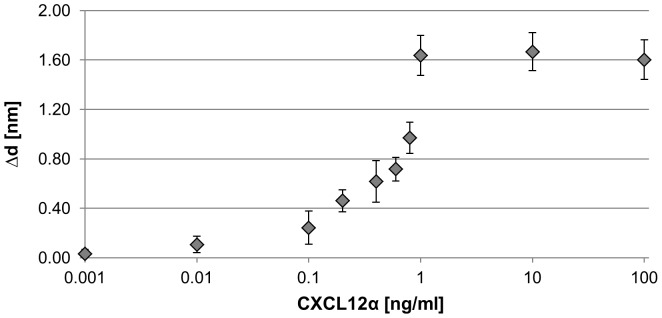
CXCL12α binding to receptors at the cell membrane of SH-SY5Y cells. Binding of the chemokine CXCL12α to neuronal cells (SH-SY5Y) was analysed by TIRE. Calibration curves (layer thickness increment Δd vs. increasing chemokine concentrations in ng/ml) show binding to native cell membranes deposited on a gold surface. n = 3. Standards errors are shown.

### Confocal Microscopy

Ishikawa cells were deposited onto Cr/Au coated glass slides via Langmuir-Schaefer films. Z-stack images of phase-contrast views were taken using a confocal microscope (Zeiss LSM510 Meta laser scanning confocal microscope) on these slides and compared to Cr/Au glass slides only. These z-stacks were displayed as 3D projections and partially at a 30° angle.

**Figure 11:**
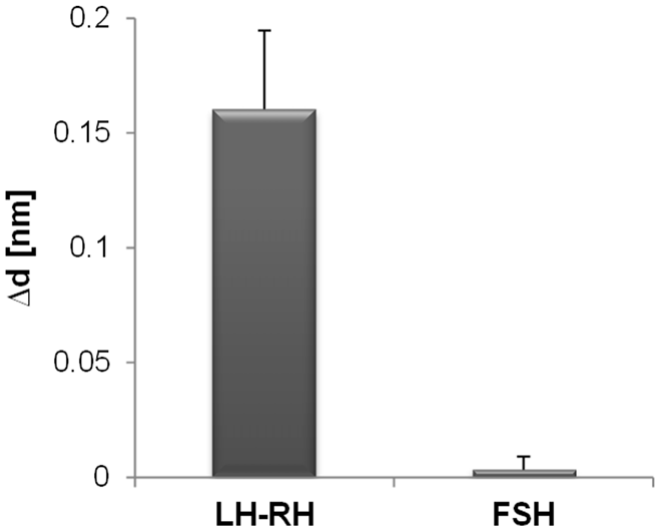
LH-LR and FSH binding to Ishikawa cells analysed by TIRE. Increase in layer thickness Δd after addition of 100 ng/ml human luteinizing hormone releasing hormone (LH-LR) is shown and compared to binding of 100 ng/ml follicle stimulating hormone (FSH) to Ishikawa cells. n = 3. Standards errors are shown.

## Results

To test the capacity of TIRE for measuring ligand-receptor interactions on native membranes a well-defined ligand-receptor couple was chosen, namely the binding of the chemokine CXCL12α to its natural membrane receptor CXCR4 and potentially CXCR7 [18] in Ishikawa endometrial adenocarcinoma cells. CXCR4 expression can be detected in Ishikawa cells at the level of both mRNA and protein, but its ligand CXCL12α is absent [27], thus allowing control over the concentration of CXCL12α for binding studies. Furthermore, CXCL12α is functional in Ishikawa cells and was shown to induce proliferation [28].

Since CXCR7 was recently described as an additional CXCL12α-binding receptor [18], [19], it was possible that it would interfere with the binding assays, so Ishikawa cells were tested for the presence of CXCR7 mRNA. Reverse transcriptase PCR was performed using primer combinations specific for CXCR7 and CXCR4, respectively. CXCR4-PCR should yield a 208-bp product, CXCR7-PCR a 189-bp product. A neuronal cell line (SH-SY5Y) was used as a control because both CXCR7 and CXCR4 are expressed in neuronal tissue [20]. CXCR4 mRNA was present in both cell lines whereas CXCR7 mRNA could only be detected in the neuronal cell line (Fig. 1). It is therefore concluded that CXCR4 is the only receptor likely to bind CXCL12α in Ishikawa cells.

In normally functioning cells, chemokine binding to their receptor causes phosphorylation at the cytosolic C-terminus of the receptor, which triggers rapid receptor internalisation [29]. If this process occurred in the TIRE assay system it would result in varying receptor levels, so this possibility was tested by immunoblotting Ishikawa cells after treatment with CXCL12α. Availability of CXCR4 did not significantly decrease after 15 min incubation with 10 ng/ml CXCL12α (Fig. 2), indicating that the Ishikawa cells prepared for TIRE analysis can be treated as a stable system for the purposes of CXCL12α binding. To check for membrane intactness Ishikawa cells frozen in Tris-buffer were thawed and spotted onto glass slides for light microscopy (Fig. 3). Although cells cannot be recultured after such freezing procedure (data not shown), probably due to bursting in the non-osmotic medium, membranes are not fragmented and hence the polarity should be intact. Taken together, the stability of receptor availability and integrity of cells indicate that the receptor is being presented in its native membrane environment for subsequent analysis of ligand binding.

To prepare Ishikawa cells for TIRE measurements they were deposited onto Cr/Au coated glass slides as a uniform layer via Langmuir-Schaefer films as shown in Figure 4. 3D confocal analysis confirmed near-saturation coverage of the Cr/Au glass slide by cells in comparison to Cr/Au glass slides only (Fig. 5). These slides were fitted on top of the ellipsometer chamber with the deposited cells facing the lumen and the chamber was flushed with the chemokine CXCL12α in increasing concentrations from 1 pg/ml to 100 ng/ml (Fig. S1). TIRE spectra were recorded in steady-state conditions after completion of adsorption of each chemokine concentration (Fig. 6). Fitting the obtained TIRE spectra allowed the evaluation of the layer thicknesses and therefore the level of binding after each adsorption step. Since ellipsometry is an indirect method the measured parameters Δ and Ψ cannot be converted directly into the optical constants of the layer sample but a model analysis must be performed. For that a layer model must be established, which considers the optical constants and thickness of all individual layers of the sample in the correct layer sequence. The refractive index is kept fixed since all bio-organic substances have similar refractive indices of approximately 1.42 at 630 nm [25]. Using least-squares minimization the thickness parameters are varied, and Δ and Ψ values are calculated using Fresnel equations. The calculated Δ and Ψ values are fitted to the experimental data and the best match provides the thickness of the layer. Here, binding was observed at concentrations as low as 100 pg/ml of CXCL12α. At a concentration of 1 ng/ml the receptors are saturated as no further increase in binding could be detected (Fig. 6). A slight decrease in layer thickness at higher concentrations is most likely due to destabilisation of the cell layer after reaching binding saturation. No binding of the proteins bovine serum albumin (BSA, 1 mg/ml) or ovalbumin (1 mg/ml) to the deposited cells could be detected (data not shown), providing further evidence of full cell coverage and a lack of unspecific binding to the charged slide.

To test whether binding of CXCL12α was due to specific interactions with membrane proteins, a proteolytic digestion of cells was optimised to selectively remove CXCR4. Cells were treated with trypsin at varying concentrations, followed by incubation with soybean trypsin inhibitor (Fig. 7). Treatment with a trypsin concentration of 1 U/µl was sufficient to reduce CXCR4 levels to below a detectable quantity (Fig. 7), so this level was chosen to test the protein-dependency of CXCL12α binding using TIRE. Cells treated with trypsin were no longer able to bind CXCL12α (Fig. 8), showing that binding is due solely to a protein component of the cells. To test whether cells are still attached to the slide after trypsin treatment layer thickness was measured before and after trypsinization; the minimal decrease in layer thickness (Δd<0.05) shows that no significant loss occurred. Furthermore, trypsinized slides did not show any binding to BSA (1 µg/ml; data not shown), which would be expected to occur in the absence of cells, providing additional evidence that the cells remain attached after trypsin treatment.

To determine the specificity of the interaction further, and to assess the capacity of the method to screen for drug candidates, an inhibitor of CXCL12α-binding to CXCR4, AMD3100, was tested. AMD3100 is a highly specific CXCR4 antagonist that has been shown to block CXCL12α action in cell-based assays and in living mice with disseminated ovarian cancer [30], and it is approved for clinical use. Mutational analysis of the CXCR4 receptor has shown that binding of AMD3100 is dependent on both Asp^171^ and Asp^262^ [31]. AMD3100 binds CXCR4 in the base of the ligand-binding pocket between Asp^171^ in TM-IV and Asp^262^ in TM-VI via its cyclam moieties, and it is suggested that AMD3100 also imposes a conformational constraint upon the receptor by the connecting phenylenebismethylene linker [31]. The TIRE cell was flushed with AMD3100 and the Ishikawa cells incubated with the drug for 10 min before flushing the cell with CXCL12α. Addition of 5 ng/ml AMD3100 reduced the ability of the ligand to bind by 65%, and a concentration of 5 µg/ml AMD3100 fully abolished ligand binding (Fig. 9), whereas AMD3100 did not cause any increase in layer thickness by itself (data not shown). This inhibition of CXCL12α binding by AMD3100 shows that the binding measurements are purely derived from the interaction between CXCR4 and CXCL12α, and that the action of small molecules can be determined on the basis of receptor-ligand interactions.

### Dynamic Measurements and Binding Kinetics

To obtain binding affinities for CXCL12α dynamic spectral measurements were carried out during molecular adsorption. Data was recorded over 10 min following flushing of the cells with the ligand. Time dependences of the phase-depending parameter Δ at 700 nm were extracted and used for the analysis of binding kinetics. Affinity constants K_A_ were calculated for chemokine concentrations from 10 pg/ml up to 10 ng/ml, representing the area of specific membrane binding (Fig. 6). The K_A_ value was determined as (1.4±0.4) x.10^9^ (l/mol), which indicates a greater binding affinity than obtained using other methods. Competitive binding assays with radiolabelled CXCL12α reported a binding affinity of the ligand to its receptor CXCR4 of K_d_ = 3.6±1.6 nM [32] corresponding to a K_A_ of 2.8×10^8^ (l/mol), and therefore five times lower than the K_A_ determined via ellipsometry. Clinical studies showed that CXCR4 is almost saturated at a CXCL12α concentration of 80 nM [32]. In the TIRE measurements binding saturation is reached at 1 ng/ml (0.13 nM) (Fig. 6), which is therefore significantly lower than previously reported. This might be due to the use of a different cell line (CEM T) and the binding inhibition assay with radiolabelled CXCL12α used in this publication. Transcriptional analysis of both cell lines, CEM T and Ishikawa cells, would be necessary to comment on comparability.

### TIRE with the Neuronal Cell Line SH-SY5Y

To show that the methodology can be used for cell lines other than Ishikawa cells, the neuronal cell line SH-SY5Y was prepared by Langmuir-Schaefer deposition and tested by TIRE. SH-SY5Y cells possess both CXCR4 and CXCR7 receptors that are capable of binding CXCL12α, as shown in Figure 3 and in previous reports [18], [19]. Binding was initially observed at 10 pg/ml (Fig. 10), and becomes saturated at a concentration of 1 ng/ml. The increase in thickness Δd is higher than in Ishikawa cells, probably due to the availability of two CXCL12α-binding receptors, and the greater affinity of CXCL12α (tenfold higher K_d_) towards CXCR7 than CXCR4 [33]. Therefore, the ability to detect ligand binding to Ishikawa cells does not appear to be due to abnormally high levels of receptor expression or other unique features of these cells.

We have shown that the chemokine CXCL12α, which is an 8 kDa peptide can be detected, but binding of the small molecule AMD3100 can only be shown indirectly via binding inhibition of CXCL12α. To investigate the size limitations of the TIRE system further, binding of the ten residue peptide human luteinizing hormone releasing hormone (LH-RH) was tested. Expression of LH-RH and its receptor has been reported in most ovarian and endometrial cancer cell lines including Ishikawa cells [34], and consistent with this, the binding of LH-RH to Ishikawa cells in TIRE could be detected at a concentration of 100 ng/ml (Fig. 11). To control for specificity of binding, the 30 kDa follicle stimulating hormone (FSH) was tested for binding to Ishikawa cells, in which its receptor is reported to be absent [35] FSH binding was not detected (Fig. 11), showing that detection by TIRE is highly specific, even though it is sensitive enough to detect the binding of small peptides.

## Discussion

Here, it has been established that TIRE can be used to quantify a specific interaction between a GPCR in its native membrane environment and its ligand. Such assays have been previously established for chloroplast receptors and their protein binding partners, but this extends the method beyond organelles to whole cells. Cells are prepared using a simple freezing technique and although such cells cannot be re-cultured –most likely due to bursting in the non-osmotic buffer system and potential major losses of soluble protein content-, membranes are not fragmented (Fig. 3) leaving the most important membrane polarity intact.

Importantly, the method should be applicable to a wide range of cells, which can be prepared using simple techniques, and will allow their interaction with external ligands to be characterised and for new binding partners to be identified.

Testing interactions at native membranes via Langmuir-Schaefer deposition and TIRE has several potential advantages over currently used techniques:

1. The high specificity and affinity of CXCR4 for CXCL12α measured using TIRE suggests that the CXCR4 is maintained in a native conformation, of which one aspect is that native CXCR4 is known to act as a homodimer [36], [37]. Furthermore, as the membranes themselves are unfragmented, it should be possible to assay receptors that depend on transient interactions or other membrane components for structural changes or interactions in their native state. Many other receptors show heterodimerization, for example GABA B receptor [38], and the function of receptors in so called “receptor mosaics” is dependent on receptor stoichiometry and topology of its individual receptors [for review 39]. Interactions can take place via transmembrane helices, domain swapping [40] or even electrostatic epitope-epitope interactions [41]. Heteromerization and receptor mosaics of GPCRs can involve allosteric mechanisms by conformational changes as shown for the α_2A_-adrenergic and μ-opioid receptors [42]. Morphine binding to the μ-opioid receptor triggers a rapid conformational change in the α_2A_-adrenergic receptor, thereby preventing overstimulation by adjusting G-protein activation. The capacity of TIRE to measure interactions in a native environment without receptor overexpression will provide significant advantages in such situations.
2. Interactions can be assessed in the native lipid environment without the need for recombinant expression of membrane proteins, which is challenging due to their structure and multiple membrane spans, purification and reconstitution. For example, nonionic detergent solubilisation of the GPCRs CXCR4 and CCR5 prevents binding of the HIV envelope protein gp120, suggesting that the lipid environment is important for receptor conformation [43].
3. Cell-specific conformations of receptors can be assessed. Conformational heterogeneity of CXCR4 in different cell backgrounds has been reported [43], based on the findings that epitope specific antibodies often show antigenic heterogeneity for CXCR4 in primary and transformed T-cell lines and isolated B cells but not in most B cell lines. Therefore testing ligand or drug interaction in the native cellular background without the need for overexpression of the receptor, which requires in most cases the use of a non-native cell line, is likely to be valuable.
4. Cotranslational modifications important for expression and function of receptors are being accounted for. CXCR4 features two potential N-linked glycosylation sites, Asn11 and Asn176 [44], within its extracellular domain. Both sites are glycosylated when CXCR4 is expressed in Sf9 insect cells [45] however, only Asn11 seems to be glycosylated in mammalian cells [46].
5. Interactions at native cell membranes account for the natural abundance of receptors. Expression levels of HIV coreceptors such as CXCR4 and CCR5 can influence virus infectivity, as shown for patients with slightly reduced CCR5, who have reduced virus loads and prolonged survival [47].

Although we have shown that binding of peptides as small as ten residues can be detected via TIRE (Fig. 11), this system will have an overall detection limit based on ligand size and receptor abundance, which in some cases can be overcome by measuring binding indirectly as shown for binding of the drug AMD3100 (Fig. 10). In these experiments the receptors are not being specifically overexpressed, and interactions with 70–90 kDa proteins have previously been detected with low abundance receptors at chloroplast membranes [12], suggesting that the sensitivity is sufficient for a wide range of membrane-based interactions.

In summary, depositing native cell membranes via Langmuir-Schaefer films displays receptor proteins in their native state, which is frequently critical for assessing their normal behaviour. Due to these advantages, potential applications of TIRE are broad-ranged, although further technique development would be required to automate the procedure for high-throughput screening. Ligands can be tested for their specificity towards different membrane and cell types, and the effect of small molecules on these interactions can be easily tested. Using dynamic scans, ligands and especially drug molecules can be modified and fine-tuned to achieve increased binding affinities. Many potential applications are apparent for investigating the function of cell membrane receptors and identifying their binding partners, which should also be useful in screens for new pharmaceuticals.

## Supporting Information

Figure S1 **Illustration of the TIRE system with Langmuir-Schaefer cell deposition.** Cr/Au coated slide with cells is placed into the ellipsometer cell which is flushed with the ligand/drug. The reflection of polarized light is measured and changes in layer thickness are detected.
(TIF)
