# Supplementary figures and images for: Quantification of Ligand Binding to G-Protein Coupled Receptors on Cell Membranes by Ellipsometry
Source: PLoS One. 2012 Sep 26;7(9):e46221. doi: 10.1371/journal.pone.0046221 (PMC3458955; doi:10.1371/journal.pone.0046221)

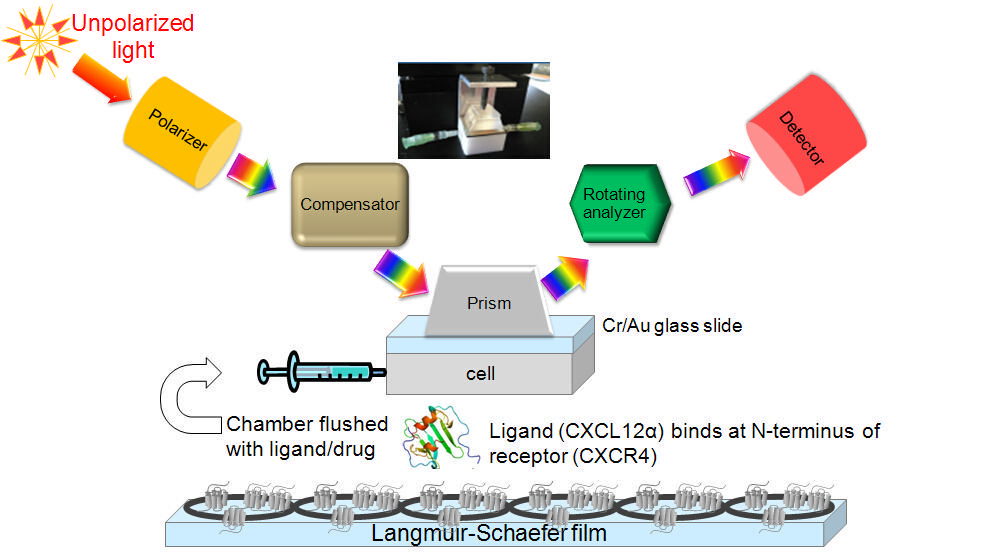

Supplement: Figure S1 — Illustration of the TIRE system with Langmuir-Schaefer cell deposition. Cr/Au coated slide with cells is placed into the ellipsometer cell which is flushed with the ligand/drug. The reflection of polarized light is measured and changes in layer thickness are detected. (TIF) [file pone.0046221.s001.tif]
